# Sparse Logistic Regression With *L*_1/2_ Penalty for Emotion Recognition in Electroencephalography Classification

**DOI:** 10.3389/fninf.2020.00029

**Published:** 2020-08-07

**Authors:** Dong-Wei Chen, Rui Miao, Zhao-Yong Deng, Yue-Yue Lu, Yong Liang, Lan Huang

**Affiliations:** ^1^School of Electronic Information Engineering, University of Electronic Science and Technology of China, Zhongshan, China; ^2^Faculty of Information Technology, Macau University of Science and Technology, Macau, China; ^3^State Key Laboratory of Quality Research in Chinese Medicines, Macau University of Science and Technology, Macau, China

**Keywords:** EEG, emotion recognition, *L*_1_ regularization, Ridge Regression, *L*_1/2_ regularization, sparse logistic regression

## Abstract

Emotion recognition based on electroencephalography (EEG) signals is a current focus in brain-computer interface research. However, the classification of EEG is difficult owing to large amounts of data and high levels of noise. Therefore, it is important to determine how to effectively extract features that include important information. Regularization, one of the effective methods for EEG signal processing, can effectively extract important features from the signal and has potential applications in EEG emotion recognition. Currently, the most popular regularization technique is Lasso (*L*_1_) and Ridge Regression (*L*_2_). In recent years, researchers have proposed many other regularization terms. In theory, *L*_*q*_-type regularization has a lower *q* value, which means that it can be used to find solutions with better sparsity. *L*_1/2_ regularization is of *L*_*q*_ type (0 < *q* < 1) and has been shown to have many attractive properties. In this work, we studied the *L*_1/2_ penalty in sparse logistic regression for three-classification EEG emotion recognition, and used a coordinate descent algorithm and a univariate semi-threshold operator to implement *L*_1/2_ penalty logistic regression. The experimental results on simulation and real data demonstrate that our proposed method is better than other existing regularization methods. Sparse logistic regression with *L*_1/2_ penalty achieves higher classification accuracy than the conventional *L*_1_, Ridge Regression, and Elastic Net regularization methods, using fewer but more informative EEG signals. This is very important for high-dimensional small-sample EEG data and can help researchers to reduce computational complexity and improve computational accuracy. Therefore, we propose that sparse logistic regression with the *L*_1/2_ penalty is an effective technique for emotion recognition in practical classification problems.

## 1. Introduction

Electroencephalography (EEG) is a means of obtaining data through sensors (Rashid et al., 2018; Uktveris and Jusas, 2018). The brain-computer interface (BCI), also known as a direct neural interface, is an interdisciplinary cutting-edge technology that represents a direct link between human or animal brains (or brain cell cultures) and external devices (Wolpaw et al., 2000, 2002; Cecotti, 2011; Chaudhary et al., 2016; Ramadan and Vasilakos, 2017). The role of BCI is to establish communication between the human brain and external computers or other intelligent electronic devices (Jin et al., 2015; Li et al., 2016). Emotional cognition is a very important part of BCI. Emotional recognition generally refers to the use of an individual's physiological or non-physiological signals to automatically identify their emotional state (Cowie et al., 2001; Busso et al., 2004). Emotional recognition is an important part of emotional computing and is of great importance in medicine and engineering.

Pattern recognition is a crucial step in accurately classifying or decoding EEG signals in BCI. How to effectively identify and classify EEG features is still the subject of research. Several EEG classification algorithms have been proposed, including logistic regression, support vector machine (Chen et al., 2019), decision tree (Subasi and Erçelebi, 2005; Polat and Güneş, 2007; Subasi and Gursoy, 2010), and convolutional neural networks (Baran-Baloglu et al., 2019; Bernal et al., 2019). These methods tend to focus on classification, and usually aim to directly find a possible classification model. The classification process does not usually involve sparse processing. However, EEG data tend to be characterized by high dimensions and small sample sizes. Therefore, these methods are prone to over-fitting or low precision. There are two main approaches to this problem. The first is dimension reduction, the main examples of which are the PCA and LDA methods (Subasi and Gursoy, 2010). These methods use matrix decomposition to map the original N-dimensional features into K dimensions, thereby changing the original values of the data. The second approach is regularization, currently represented by the *L*_0_, Lasso (*L*_1_), Ridge Regression, and Elastic Net methods (Silva et al., 2004; Zou and Hastie, 2005; Friston et al., 2008; Wang et al., 2015). In theory, the *L*_0_ penalty is the best in the case of sparseness, but this method involves an NP-hard problem (Schölkopf and Smola, 2001). Therefore, the Lasso (*L*_1_) penalty is most often used. The *L*_1_ penalty is the sum of the absolute values of the elements in the weight vector *w*, usually expressed as ||ω||_1_ (Tibshirani, 1996). The Ridge Regression is the sum of the squares of the elements in the weight vector *w* and the square root, usually expressed as ||ω||_2_ (Ng, 2004). The Elastic Net method was proposed to overcome the respective limitations of *L*_1_ and Ridge Regression. This method combines the *L*_1_ penalty and Ridge Regression to achieve a better effect. Recently, in order to obtain a sparser and more solvable penalty term, Xu proposed a new *L*_1/2_ penalty and applied it to signal recovery problem (Xu et al., 2012); the result of the this penalty is more sparse than that of the *L*_1_ penalty and can be solved. It is thus preferable in theory (Xu et al., 2010).

In the field of multi-category EEG emotion recognition, sparse logistic regression models based on regularization have achieved excellent results in EEG signal emotion recognition in recent years. For example, Ryali et al. (2010) proposed a novel method based on logistic regression using a combination of *L*_1_ and Ridge Regression that could more accurately discriminate brain regions across multiple conditions or groups (Ryali et al., 2010). Hussein et al. (2018) proposed a feature learning method based on *L*_1_-penalized robust regression, which could recognize the most prominent features pertinent to epileptic seizures in EEG spectra (Hussein et al., 2018). Conroy and Sajda (2012) used Ridge Regression to improve EEG classification results (Conroy and Sajda, 2012). Inspired by the above methods, we studied sparse logistic regression models with the *L*_1/2_ penalty, with a particular focus on applications in EEG sentiment classification. The *L*_1/2_ penalty can be penalized as representatives of (0 < *q* < 1) and has many attractive features, such as unbiasedness, sparsity, and oracle attributes (Xu et al., 2010). Current logistic regression models using the *L*_1/2_ penalty have achieved excellent results in biological fields, such as genetic screening (Liang et al., 2013; Liu et al., 2014; Huang et al., 2016).

**Figure 1:**
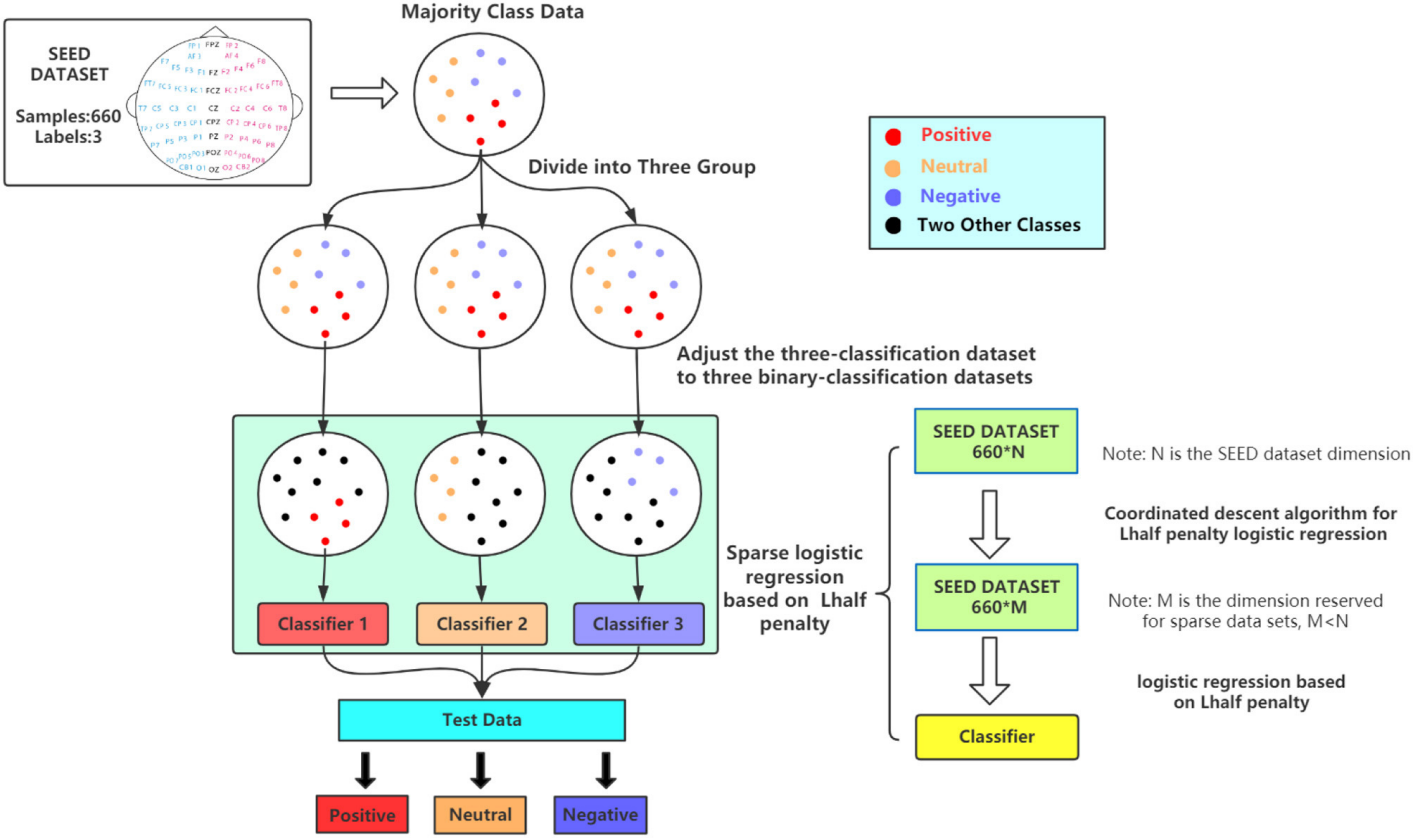
Flow chart of sparse logistic regression based on *L*_1/2_ penalty. First, we divide a three-category dataset (such as SEED) into three two-category datasets (the upper part). Next, the green part represents training a classifier based on *L*_1/2_ penalty logistic regression for each binary classification dataset. Each classifier will reduce the sample dimension from N to M after sparsification. For each input EEG data, we will get three binary classification results. Through summary analysis, the final three classification results are obtained.

In this work, we develop a coordinate reduction algorithm for *L*_1/2_ regularization in a sparse logistic regression framework and build a three-classification sparse regularization logistic regression model for EEG sentiment data (Figure 1). This method is suitable for use with large EEG datasets with a low sample size. Tests were performed using a simulation dataset and a real dataset (SEED and DEAP). An experimental comparison with sparse logistic regression using the *L*_1_ penalty, Ridge Regression, and Elastic Net penalty points was used to validate the *L*_1/2_ penalty logistic regression method proposed in this paper.

## 2. Materials and Methods

### 2.1. Materials

#### 2.1.1. Simulation Dataset

We used the Python method sklearn.dataset.make_classification to generate a random simulation three-classification dataset, with a sample number of 1,200 and a characteristic of 1,000. Each class is composed of a number of gaussian clusters each located around the vertices of a hypercube in a subspace of dimension 3. For each cluster, informative features are drawn independently from *N*(0, 1) (Equation 1) and then randomly linearly combined within each cluster in order to add covariance. The clusters are then placed on the vertices of the hypercube. And we added useless features drawn at random to this dataset use parameters *n*_*redundant* = 2 and *n*_*repeated* = 2 to add two redundant features to the information feature and repeated it twice. We selected 80% of the samples as the training dataset and used the remaining 20% as the verification dataset.

(1) $$\begin{array}{r} f\left(x\right)=\frac{1}{\sqrt{2\pi }}e^{\left(-\frac{x^{2}}{2}\right)} \end{array}$$

#### 2.1.2. SEED Dataset

Experiments were conducted using a public emotion EEG dataset called SEED, which uses film fragments as emotion-inducing materials and includes three categories of emotion: positive, neutral, and negative. In each experiment, the participants will watch movie clips of different emotional states. Each clip will be played for about 4 min. In the experiment, three types of movie clips will be played. Each type of movie clips contains five movies, and in total 15 movies. These movie clips are all from Chinese movies. There is a 5 s prompt before each short film is shown, with 45 s of feedback time after playback, and 15 s of rest after watching. A total of 15 subjects (seven males, eight females, mean age 23.27 years old, the standard deviation of 2.37) participated in the experiment, and all subjects had normal visual, auditory, and emotional states. The EEG signal, while the subject was watching the movie, was recorded through the electrode cap and the sampling frequency was 1,000 Hz. The experiment used the international 10–20 system and a 62-channel electrode cap. Each volunteer participated in three experiments, and each experiment was separated by about 1 week. Therefore, a total of 15 × 15 × 3 = 675 data samples is formed. Then 200 Hz down-sampling and a bandpass frequency filter from 0 to 75 Hz was applied to obtain a preprocessed EEG dataset. For more information on this dataset, please refer to the website http://bcmi.sjtu.edu.cn/~seed/index.html. Before using this dataset, we used the PCA method to preprocess the data and to reduce the dimension from 11,470 to 528 in the beta band, and from 57350 to 528 in the combined band (keep 95% variance information), in order to reduce the computational complexity and time complexity (according to the results of running the PCA model based on the SEED dataset, the variance ratio of the first PC is only 30.1%, the variance ratio of the second PC is 9.2%, the variance ratio of the third PC is only 4.9%, and the sum of the variance ratios of the three largest contributing PCs is only 44.2%, which is much <95% variance ratio required in this paper. In this situation, program results show that we need 528 PCs to achieve a 95% variance ratio).

##### 2.1.2.1. Beta band dataset

The SEED EEG dataset contained five EEG bands. The main frequency range of the five bands was 14–30 Hz. The frequency range of the beta brain wave is 14–30 Hz. When the brain is in a conscious condition, the mind is in a state of tension, and the individual is very sensitive to their surroundings, so the energy intensity of the beta wave will be higher than the others. The attention is focused on the external environment in a scattered manner, and the brain is prone to fatigue. Most people are in this state during the day. Previous studies have shown that the main role of the beta band is to reflect emotions and cognition (Ahmed and Basori, 2013; Jabbic et al., 2015). Therefore, we chose beta brain waves for experimental analysis. The beta brain wave frequency band of the SEED dataset contained 660 samples.

##### 2.1.2.2. Combined band dataset

In order to verify the performance of our method, we also tested it using the EEG dataset for the total frequency band. The EEG signal is decomposed into five frequency bands according to EEG rhythm, comprising delta (1–3 Hz), theta (4–7 Hz), alpha (8–13 Hz), beta (14–30 Hz), and gamma (31–50 Hz) bands. These five frequency band signals were combined to form a new combined frequency band dataset (Lin et al., 2010; Nie et al., 2011). Therefore, six EEG datasets representing different frequency bands were obtained. Finally, four classification methods, namely sparse logistic regression with *L*_1/2_ penalty, sparse logistic regression with *L*_1_ penalty, Ridge Regression, and Elastic Net, were tested and verified using the above datasets. In the experiments, 660 samples were randomly assigned to the mutually exclusive training set (80%) and the remainder formed the verification set (20%).

#### 2.1.3. DEAP Dataset

The dataset named DEAPA Database for Emotion Analysis Using Physiological Signals (Koelstra et al., 2012) can be found at the website http://www.eecs.qmul.ac.uk/mmv/datasets/deap/. The DEAP dataset consists of two parts; first the ratings from an online self-assessment where 120 1-min extracts of music videos were each rated by 14–16 volunteers based on arousal, valence and dominance; second, the participant ratings, physiological recordings and face video of an experiment where 32 volunteers watched a subset of 40 of the above music videos. EEG and physiological signals were recorded, and each participant also rated the videos as above. For 22 participants frontal face video was also recorded. At the end of each video, participants are required to fill out a self-assessment (SAM) form to score from 1 to 9. Arousal ranges from inactive (1) to active (9). Valence ranges from unpleasant (1) to pleasant (9). The rating range of liking and dominance is also between 1 and 9, which means helpless and a weak feeling (1) to an empowered feeling (9). The DEAP dataset includes 32-channel EEG signals, and peripheral physiological signals, such as GSR signals, EOG signals, EMG signals, PPG signals, Temp, and Status. All data was down-sampled to 128 Hz, where the EEG signal data became a 60 s test signal and a 3 s baseline. A zero-phase bandpass filter of 4–45 Hz was applied. In this paper, the 32-channel EEG was divided into two classes according to arousal, Positive (more than 6) and Negative (Low 4). For the DEAP dataset, we only used data from combined frequency bands for experiments.

#### 2.1.4. Cross-Validation

To ensure the accuracy of the results, a 5-fold cross-validation method was used in all the experiments. The 5-fold cross-validation first divides all the data into five sub-samples. One of the sub-samples is repeatedly selected as the test set, and the other four samples are used for training, repeated five times in total and the average of five times and its error range is selected. In addition to using 5-fold cross-validation, all experiments in this paper also performed 100 repeated experiments to obtain its averages and errors.

### 2.2. Methods

This paper constructs a three-category *L*_1/2_ penalty logistic regression method. The main focus of this work was the general ternary classification problem. A ternary classifier was built, consisting of three small two classifiers, each of which was identical in construction. This model produced three two-classifier results, which could be summarized to give the ternary classification output, as shown in Figure 1. We first summarize a three-category dataset into three two-category datasets, and then establish three two-category *L*_1/2_ penalty logistic regression methods. According to the results of each two-classifier, the final three-classification result is obtained. The construction method of the *L*_1/2_ penalty logistic regression method for the two classifications is as follows.

#### 2.2.1. Sparse Logistic Regression With *L*_1/2_ Penalty

Here, we describe the construction of a sparse two-class logistic regression method based on the *L*_1/2_ penalty. Suppose we have *n* samples, where *D* = (*X*_1_, *y*_1_), (*X*_2_, *y*_2_), ..., (*X*_*n*_, *y*_*n*_), *X*_*i*_ = (*x*_*i*1_, *x*_*i*2_, ..., *x*_*ip*_) is the *i*th input mode, and the dimension is *p*; *y*_*i*_ is the corresponding variable, with a value of 0 or 1: *y*_*i*_ = 0 represents the *i*th sample in category 1, and *y*_*i*_ = 1 represents the *i*th sample in category 2. Vector *X*_*i*_ includes the *p* features of the *i*th samples (for all *p* EEG signals), and *x*_*ij*_ represents the EEG signal value of *j* in the *i*th sample. Defining the classifier as *f*(*x*) = *e*^*x*^/(1 + *e*^*x*^) allows *y* to be correctly predicted using the class label *y* for any input *x*.

The logistic regression is expressed as:

(2) $$\begin{array}{r} P\left(y_{i}=1|X_{i}\right)=f\left({X^{\prime }}_{i}\beta \right)=\frac{exp\left({X^{\prime }}_{i}\beta \right)}{1+exp\left({X^{\prime }}_{i}\beta \right)} \end{array}$$

where β = (β_0_, β_1_, ..., β_*p*_) is the estimated coefficient, and note is the intercept. The log likelihood is

(3) $$\begin{array}{r} l\left(\beta |D\right)=-\overset{n}{\underset{i=1}{\sum }}\left\{y_{i}log\left[f\left({X^{\prime }}_{i}\beta \right)\right]+\left(1-y_{i}log\left[1-f\left({X^{\prime }}_{i}\beta \right)\right]\right)\right\}. \end{array}$$

We obtain β by minimizing the log likelihood. In high-dimensional applications with *p* >> *n*, directly solving the logical model given in Equation (3) is ill-posed and may lead to over-fitting. Therefore, it is necessary to apply a regularization method to solve the over-fitting problem. When adding a regularization term to Equation (3), the sparse logistic regression can be modeled as:

(4) $$\begin{array}{r} \beta =argmin\left\{l\left(\beta |D\right)+\lambda \overset{p}{\underset{j=1}{\sum }}P\left(\beta_{i}\right)\right\}, \end{array}$$

where λ > 0 is an adjusting parameter and *P*(β) is a regularization item. The most popular regularization technique is Lasso (*L*_1_) (Tibshirani, 1996), which uses the regularization term *P*(β) = ∑|β|. In recent years, many *L*_*q*_-type regularization terms have been proposed, including SCAD (Shailubhai et al., 2000), Elastic Net (Maglietta et al., 2007), and MC+ (Wiese et al., 2007).

In theory, *L*_*q*_-type regularization *P*(β) = ∑|β|^*q*^ with a lower *q* value will result in a better solution with more sparsity, such as signal recovery problem and genetic selection (Xu et al., 2010, 2012; Liang et al., 2013). However, when *q* is very close to zero, convergence may be difficult. Therefore, Xu et al. (2010) further explored the nature of *L*_*q*_(0 < *q* < 1) regularization and revealed the importance and effects of *L*_1/2_ regularization. They proposed that when $\frac{1}{2}<q<1$, *L*_1/2_ regularization produces the most sparse results, with relatively easy convergence compared with *L*_1_ regularization. At $0<q<\frac{1}{2}$, there is no significant difference in the performance of the *L*_*q*_ penalty. Furthermore, solving *L*_1/2_ normalization is much simpler than solving *L*_0_ normalization. Therefore, *L*_1/2_ regularization can be used as a representative of *L*_*q*_(0 < *q* < 1) regularization. In this paper, we apply the *L*_1/2_ penalty to the logistic regression model. A sparse logistic regression model based on the *L*_1/2_ penalty has the following form:

(5) $$\begin{array}{r} \beta_{1/2}=argmin\left\{l\left(\beta |D\right)+\lambda \overset{p}{\underset{j=1}{\sum }}|\beta_{j}{|}^{\frac{1}{2}}\right\}. \end{array}$$

*L*_1/2_ regularization has been shown to have many attractive features, including unbiasedness, sparsity, and oracle features (Xu et al., 2010, 2012; Liang et al., 2013). Theoretical and experimental analyses show that regularization is a competitive approach. Our work in this paper also reveals the effectiveness of *L*_1/2_ regularization in solving non-linear logistic regression problems with a small number of predictive features (EEG signals).

#### 2.2.2. Coordinated Descent Algorithm for *L*_1/2_ Penalty Logistic Regression

The coordinate descent algorithm (Friedman et al., 2007, 2010) is a “single-at-time” method. Its basic steps can be described as follows: for each coefficient, the remaining elements are partially fixed relative to the optimization objective function β_*j*_(*j* = 1, 2, ..., *p*) in the nearest updated value.

Before introducing the coordinate reduction algorithm for non-linear logistic regularization, we first consider the linear regularization case. Assuming that dataset D has *n* samples, *D* = (*X*_1_, *y*_1_), (*X*_2_, *y*_2_), ..., (*X*_*n*_, *y*_*n*_), where *X*_*i*_ = (*x*_*i*1_, *x*_*i*2_, ..., *x*_*ip*_) is the *i*th input variable, the dimension is *p*, and *y*_*i*_ is the corresponding response variable. Variables are standardized: $\overset{n}{\underset{i=1}{\sum }}{x_{ij}}^{2}=1$ and $\overset{n}{\underset{i=1}{\sum }}y_{i}=0$. Therefore, the linear regression of the regularization term can be expressed as:

(6) $$\begin{array}{r} R\left(\beta \right)=argmin\left\{\frac{1}{n}\overset{n}{\underset{i=1}{\sum }}{\left(y_{i}-X^{\prime }\beta \right)}^{2}+\lambda \overset{p}{\underset{j=1}{\sum }}P\left(\beta_{j}\right)\right\}, \end{array}$$

where *P*(β) is the regularization term. The coordinate descent algorithm can be used to solve β_*j*_, and the other β_*k*≠*j*_ (*k* ≠ *j* represents the parameters that remain after *j*th the element is removed) are fixed. According to the idea of coordinate descent algorithm, only one variable is optimized at each time and other variables are fixed. The function can be expressed as

(7) $$\begin{array}{r} F\left(\beta \right)=\frac{1}{n}{\left(y_{i}-\underset{k\neq j}{\sum }x_{ik}\beta_{k}+x_{ij}\beta_{j}\right)}^{2}+\lambda \underset{k\neq j}{\sum }P\left(\beta_{k}\right)+\lambda P\left(\beta_{j}\right). \end{array}$$

The first derivative β_*j*_ can be estimated as:

(8) $$\begin{array}{r} \frac{\partial R}{\partial \beta_{j}}=\overset{n}{\underset{i=1}{\sum }}\left(-x_{ij}\left(y_{i}-\underset{k\neq j}{\sum }x_{ik}\beta_{k}-x_{ij}\beta_{j}\right)\right)+\lambda P{\left(\beta_{j}\right)}^{\prime }=0. \end{array}$$

Defining ${\tilde{y_{i}}}^{\left(j\right)}=\underset{k\neq j}{\sum }x_{ik}\beta_{k}$ as the partial residual for fittingβ_*j*_ and $\omega_{j}=\overset{n}{\underset{i=1}{\sum }}x_{ij}\left(y_{i}-{\tilde{y_{i}}}^{\left(j\right)}\right)$, the univariate soft thresholding operator of the coordinate descent algorithm (Busso et al., 2004) for *L*_1_ regularization (Lasso) can be defined as:

(9) $$\begin{array}{l} \beta_{j}=S\left(\omega_{j},\lambda \right)=\{\begin{array}{ll} \omega_{j}+\lambda & \text{if }\omega_{j}<-\lambda \\ \omega_{j}-\lambda & \text{if }\omega_{j}>\lambda \\ 0 & \text{if }|\omega_{j}|<\lambda \end{array}. \end{array}$$

Similarly, for *L*_0_ regularization, the threshold operator of the coordinate descent algorithm can be defined as:

(10) $$\begin{array}{r} \beta_{j}=Half\left(\omega_{j},\lambda \right)=\omega I\left(|\omega_{j}|>\lambda \right), \end{array}$$

where *I* is the indicator function. This formula is equivalent to the hard thresholding operator (Silva et al., 2004).

According to Equations (9) and (10), different penalties are associated with different threshold operators. Therefore, Xu et al. (2012) proposed a semi-threshold operator to solve the *L*_1/2_ regularization of linear regression models, using an iterative algorithm that can be considered a multivariate half-threshold method. In this paper, we present a univariate half-threshold operator for the coordinate reduction algorithm for *L*_1/2_ regularization. Based on Equation (8), the gradient of *L*_1/2_ regularization at β_*j*_ can be expressed as:

(11) $$\begin{array}{r} \frac{\partial R}{\partial \beta_{j}}=\beta_{j}-\omega_{j}+\lambda \frac{sign\left(\beta_{j}\right)}{4\sqrt{\left|\beta_{j}\right|}}=0, \end{array}$$

where β_*j*_ > 0 and $\sqrt{|\beta_{j}|=\mu },\beta_{j}=\mu^{2}$. When β_*j*_ > 0, the equation (11) can be redefined as:

(12) $$\begin{array}{r} \mu^{3}-\omega_{j}\mu +\frac{\lambda }{4}=0. \end{array}$$

A univariate half-threshold operator can be expressed as:

(13) $$\begin{array}{l} \beta_{j}=Half\left(\omega_{j},\lambda \right)=\{\begin{array}{ll} \frac{2}{3}\omega_{j}\left(1+cos\left(\frac{2\left(\pi -\phi_{\lambda }\left(\omega_{j}\right)\right)}{3}\right)\right) & \text{if}|\omega_{j}|>\frac{3}{4}{\left(\lambda \right)}^{\frac{2}{3}}\\ 0 & otherwise, \end{array} \end{array}$$

where ϕ_λ_(ω) satisfies:

$$cos\left(\phi_{\lambda }\left(\omega \right)\right)=\frac{\lambda }{8}{\left(\frac{\left|\omega \right|}{3}\right)}^{-\frac{2}{3}}.$$

The *L*_1/2_ regularized coordinate reduction algorithm reuses the univariate half-threshold operator. This coordinate descent algorithm for regularization can be extended to sparse logistic regression models. Based on the objective function of sparse logistic regression (Equation 4), a Taylor series expansion of *l*(β) has the following formula:

(14) $$\begin{array}{r} L\left(\beta ,\lambda \right)\approx \frac{1}{2n}\overset{n}{\underset{i=1}{\sum }}{\left(Z_{i}-X_{i}\beta \right)}^{\prime }W_{i}\left(Z_{i}-X_{i}\beta \right)+\overset{p}{\underset{j=1}{\sum }}P\left(\beta_{j}\right), \end{array}$$

where $Z_{i}=X_{i}\tilde{\beta }+\frac{Y_{i}-f\left(X_{i}\tilde{\beta }\right)}{f\left(X_{i}\tilde{\beta }\right)\left(1-f\left(X_{i}\tilde{\beta }\right)\right)}$ is an estimated response, $W_{i}=f\left(X_{i}\tilde{\beta }\right)\left(1-f\left(X_{i}\tilde{\beta }\right)\right)$ is a weight, and $f\left(x_{i}\tilde{\beta }\right)=\frac{exp\left(X_{i}\tilde{\beta }\right)}{\left(1+exp\left(X_{i}\tilde{\beta }\right)\right)}$ is a value evaluated using the current parameters. Redefining the partial residual for fitting the current $\tilde{\beta }$ as:

(15) $$\begin{array}{r} {\tilde{Z}}_{i}^{\;(j)}=\sum_{i=1}^{n}W_{i}({\tilde{Z}}_{i}-\sum_{k\neq j}x_{ik}{\tilde{\beta }}_{k}), \end{array}$$

(16) $$\begin{array}{r} \overset{n}{\underset{i=1}{\sum }}x_{ij}\left(Z_{i}-{\tilde{Z}}_{i}^{\;(j)}\right), \end{array}$$

we can directly apply the coordinate descent algorithm with the *L*_1/2_ penalty for sparse logistic regression.

## 3. Results

Sparse logistic regression with the *L*_1/2_, *L*_1_ penalties, and Ridge Regression and the Elastic Net method were tested using the simulated dataset and the real dataset. Four evaluation methods were used to evaluate the performance of the proposed model. A confusion matrix was used to compare the results between the various methods. This is a situation analysis table that summarizes the prediction results of a classification model in machine learning. The records in the dataset are summarized in matrix form according to the real category and the classification criteria predicted by the classification model. The rows of the matrix represent the true values, and the columns represent the predicted values. The computational accuracy of the proposed method was also used as a measure of quality, where the accuracy is defined as the ratio of the number of samples correctly classified by the classifier to the total number of samples in the test dataset. However, accuracy is not always effective for performance evaluation, especially if the number of samples with different labels are not exactly equal. Therefore, we also analyzed precision and recall for further comparison of the three two-classifiers. Here, precision refers to the proportion of all predicted true positives in positive classes, and recall refers to the proportion of positives found in all positive classes. All experiments used 5-fold cross-validation to ensure the stability of the proposed model.

### 3.1. Analysis of Simulation Dataset

We used sparse logistic regression with the *L*_1/2_, *L*_1_ penalties, and Ridge Regression and Elastic Net for experiments and comparisons. Table 1 shows the accuracy and recall results for the three categories and four methods using the simulation dataset.

**Table 1:** Precision and recall results for sparse logistic regression with *L*_1/2_ and *L*_1_ penalties, Ridge Regression and Elastic Net.

**Method**	**Neutral**		**Positive**		**Negative**	
	**Precision**	**Recall**	**Precision**	**Recall**	**Precision**	**Recall**
*L*_1/2_	**1.00** ± **0.00**	**1.00** ± **0.00**	**0.99** ± **0.01**	**1.00** ± **0.00**	**1.00** ± **0.00**	**0.98** ± **0.02**
*L*_1_	0.98 ± 0.006	0.99 ± 0.02	0.97 ± 0.01	1.00 ± 0.01	0.98 ± 0.02	0.92 ± 0.03
Ridge Regression	0.92 ± 0.01	0.84 ± 0.02	0.84 ± 0.09	0.85 ± 0.08	0.63 ± 0.01	0.69 ± 0.03
Elastic Net	0.98 ± 0.01	0.99 ± 0.01	0.94 ± 0.02	1.00 ± 0.00	0.98 ± 0.02	0.88 ± 0.01

*Neutral, Positive and Negative are Emotional categories. Precision and Recall are are the criteria for judging the quality of the method. Numbers indicate the best of the four results*.

As shown in Table 1, in terms of precision, the sparse logistic regression method with the *L*_1/2_ penalty proposed in this paper was superior to the other methods in all three categories. For neutral emotion, the precision of the proposed method was 100%, which was 2, 2, and 8% higher than those of the Elastic Net method, sparse logistic regression with *L*_1_ penalty, and Ridge Regression, respectively. For positive emotion, the precision of the proposed method was 99%, which was 5, 2, and 15% higher than those of the Elastic Net method, sparse logistic regression with *L*_1_ penalty, and Ridge Regression, respectively. For negative emotion, the precision of the proposed method was 100%, which was 2, 2, and 37% higher than those of the Elastic Net method, sparse logistic regression with *L*_1_ penalty, and Ridge Regression, respectively. In terms of recall rate and precision rate, the results for sparse logistic regression with *L*_1/2_ penalty were better than those of the other three methods for positive emotion, negative emotion, and neutral emotion. For neutral emotion, the recall rate of the proposed method was 100%, which was 1, 1, and 16% higher than those of the Elastic Net method, sparse logistic regression with *L*_1_ penalty, and Ridge Regression, respectively. For positive emotion, the recall rate of the proposed method was 100%, which was 15% higher than that of the sparse logistic regression method with Ridge Regression, and the same as those of the Elastic Net method and sparse logistic regression with *L*_1_ penalty. For negative emotion, the recall rate of the proposed method was 98%, which was 10, 6, and 29% higher than those of the Elastic Net method, sparse logistic regression with *L*_1_ penalty, and Ridge Regression, respectively. Overall, the experimental results show that sparse logistic regression with the *L*_1/2_ penalty is superior to the three existing regularization methods.

**Figure 2:**
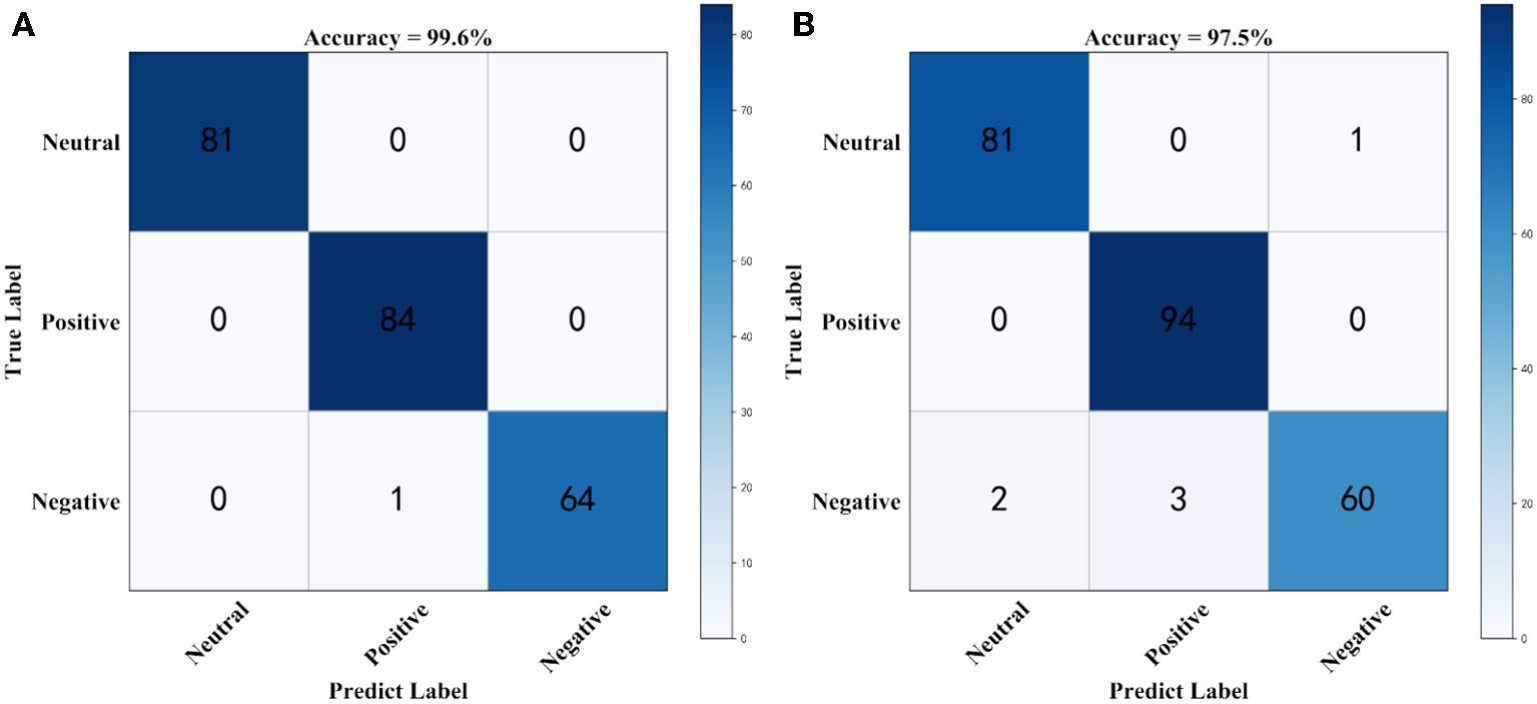
The confusion matrix using simulation dataset. **(A)** Confusion matrix for generating prediction results using sparse logistic regression with *L*_1/2_ penalty, **(B)** Confusion matrix for generating prediction results using sparse logistic regression with *L*_1_ penalty.

Figure 2 shows the confusion matrix for generating prediction results using sparse logistic regression with the *L*_1/2_ and *L*_1_ penalties for simulation datasets. As shown in Figure 2, the accuracy of the sparse logistic regression method with *L*_1/2_ penalty was 99.6%, and The results of the proposed method were significantly better than those obtained using sparse logistic regression with *L*_1_ penalty or Ridge Regression, or the Elastic Net method (Supplementary Figure 1). In the simulated dataset, there is only one label that was predicted incorrectly, using the sparse logistic regression method with *L*_1/2_ penalty. However, there are six labels that were predicted incorrectly using the sparse logistic regression method with *L*_1_ penalty. Thus, sparse logistic regression with the *L*_1/2_ penalty had the highest classification accuracy and the best effects, indicating the superiority of this method in terms of accuracy for the classification of datasets. Next, we tested our method using a real EEG emotion dataset.

### 3.2. Analysis of Beta Band Dataset

Sparse logistic regression with the *L*_1/2_, *L*_1_ penalties, and Ridge Regression and Elastic Net were used for all experiments and comparisons. Table 2 shows the accuracy and recall for the three categories using the four methods for this dataset.

**Table 2:** Precision and recall results for sparse logistic regression with *L*_1/2_ and *L*_1_ penalties, Ridge Regression and Elastic Net for the beta band dataset.

**Method**	**Neutral**		**Positive**		**Negative**	
	**Precision**	**Recall**	**Precision**	**Recall**	**Precision**	**Recall**
*L*_1/2_	**0.80** ± **0.01**	**0.71** ± **0.01**	**0.74** ± **0.02**	**0.81** ± **0.03**	**0.77** ± **0.02**	**0.81** ± **0.04**
*L*_1_	0.74 ± 0.01	0.68 ± 0.02	0.67 ± 0.01	0.72 ± 0.03	0.72 ± 0.02	0.74 ± 0.03
Ridge Regression	0.73 ± 0.03	0.70 ± 0.04	0.69 ± 0.02	0.67 ± 0.01	0.67 ± 0.01	0.71 ± 0.02
Elastic Net	0.76 ± 0.01	0.66 ± 0.04	0.71 ± 0.01	0.67 ± 0.01	0.64 ± 0.04	0.76 ± 0.02

*Neutral, Positive and Negative are Emotional categories. Precision and Recall are are the criteria for judging the quality of the method. Numbers indicate the best of the four results*.

As shown in Table 2, in terms of precision, the sparse logistic regression method with *L*_1/2_ penalty proposed in this paper was superior to other methods in all three categories. For neutral emotion, the precision of the proposed method was 80%, which was 4, 6, and 7% higher than those of the Elastic Net method, and sparse logistic regression with *L*_1_ penalty and Ridge Regression, respectively. For positive emotion, the precision of the proposed method was 74%, which was 3, 7, and 5% higher than those of the Elastic Net method, and sparse logistic regression with *L*_1_ penalty and Ridge Regression, respectively. For negative emotion, the precision of the proposed method was 77%, which was 13, 5, and 10% higher than those of the Elastic Net method, and sparse logistic regression with *L*_1_ penalty and Ridge Regression, respectively. In terms of recall rate and precision rate, the results of the sparse logistic regression method with *L*_1/2_ penalty were better than those of the other three methods for positive emotion, negative emotion, and category 3. For neutral emotion, the recall rate of the proposed method was 70%, which was 4 and 2% higher than those of Elastic Net method and sparse logistic regression with *L*_1_ penalty, respectively, and the same as that of Ridge Regression. For positive emotion, the recall rate of the proposed method was 81%, which was 14, 9, and 14% higher than those of the Elastic Net method, and sparse logistic regression with *L*_1_ penalty and Ridge Regression, respectively. For negative emotion, the recall rate of the proposed method was 81%, which was 5, 7, and 10% higher than those of the Elastic Net method, and sparse logistic regression with *L*_1_ penalty and Ridge Regression, respectively. Overall, the experimental results show that the sparse logistic regression method with *L*_1/2_ penalty is superior to the three existing regularization methods.

Supplementary Figure 2 shows an accuracy box plot for the different methods obtained using 5-fold cross-validation. It can be seen from the box plot that the results for the sparse logistic regression method with *L*_1/2_ penalty were significantly better than those of the other three methods.

**Figure 3:**
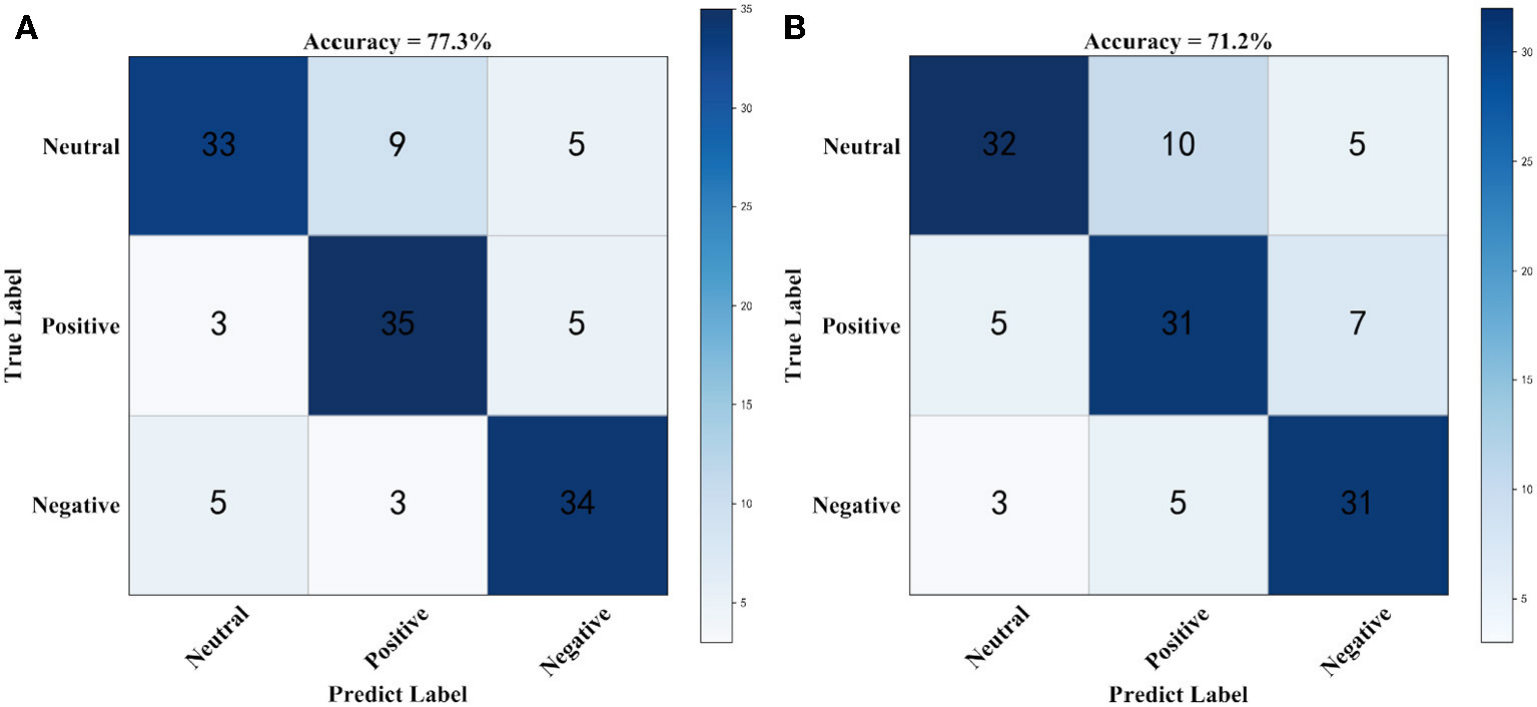
The confusion matrix using beta band dataset. **(A)** Confusion matrix for generating prediction results using sparse logistic regression with *L*_1/2_ penalty, **(B)** Confusion matrix for generating prediction results using sparse logistic regression with *L*_1_ penalty.

Figure 3 shows the confusion matrix for generating prediction results using sparse logistic regression with the *L*_1/2_ and *L*_1_ penalties for the two datasets. As shown in Figure 3, the accuracy of sparse logistic regression with *L*_1/2_ penalty was 77.3%, and the results obtained with the proposed method were significantly better than those for sparse logistic regression with the *L*_1_ penalty or Ridge Regression or the Elastic Net method (Supplementary Figure 3). In the SEED Beta band dataset, there were 30 labels that were predicted incorrectly using the sparse logistic regression method with *L*_1/2_ penalty with the main errors concentrated in the Neutrals class. However, compared with the sparse logistic regression method with *L*_1/2_ penalty, the number of incorrect labels is 5 fewer. It can be concluded that sparse logistic regression with the *L*_1/2_ penalty has the highest classification accuracy and the best effects, indicating that this method is more accurate for the classification of datasets.

In order to verify the sparsity of the *L*_1/2_ penalty logistic regression in EEG sentiment dataset. This paper counts the number of data points retained after different Sparse methods are run. As can be seen from the Table 3, in the SEED-band data set, the *L*_1/2_ penalty retains the least number of points. In the three binary classifiers, only 125 data points are retained, while the *L*_1_ method retains 252, 199, and 271 data points, respectively. Ridge regression and Elastic Net retained all data points. This verifies that in the EEG sentiment dataset, the *L*_1/2_ penalty used in this paper has the best sparsity.

**Table 3:** The results of Number of points retained after sparsification by the sparse Logistic regressions with *L*_1/2_, *L*_1_ penalties, Ridge Regression, and Elastic Net in beta band dataset.

**Method**	**Classifier 1**	**Classifier 2**	**Classifier 3**
*L*_1/2_	**125**	**125**	**125**
*L*_1_	252	199	271
Ridge Regression	410	410	410
Elastic Net	410	410	410

*Neutral, Positive and Negative are Emotional categories. Precision and Recall are are the criteria for judging the quality of the method. Numbers indicate the best of the four results*.

### 3.3. Analysis of Combined Band Dataset

As shown in Table 4, in terms of precision, the sparse logistic regression method with *L*_1/2_ penalty proposed in this paper was superior to the other methods in all three categories. For neutral emotion, the precision of the proposed method was 85%, which was 4, 3, and 2% higher than those of the Elastic Net method, sparse logistic regression with *L*_1_ penalty, and Ridge Regression, respectively. For positive emotion, the precision of the proposed method was 90%, which was 2% higher than that of sparse logistic regression with *L*_1_ penalty or Ridge Regression, and the same as that of Elastic Net method. For negative emotion, the precision of the proposed method was 86%, which was 8 and 11% higher than those of the Elastic Net method and Ridge Regression, and the same as that of sparse logistic regression with *L*_1_ penalty. In terms of recall rate and accuracy rate, the results of the sparse logistic regression method with *L*_1/2_ penalty were better than those of the other three methods for positive emotion, negative emotion, and category 3. For neutral emotion, the recall rate of the proposed method was 87%, which was 2 and 4% higher than those of the Elastic Net method and sparse logistic regression with *L*_1_ penalty, respectively, and the same as that of Ridge Regression. For positive emotion, the recall rate of the proposed method was 88%, which was 7% higher than that of the Elastic Net method, and the same as those of the sparse logistic regression methods with *L*_1_ penalty and Ridge Regression. The greatest differences between methods were seen in the negative emotion category, where the recall rate of the proposed method was 86%, which was 3, 7, and 7% higher than those of the Elastic Net method, and the sparse logistic regression methods with *L*_1_ penalty and Ridge Regression, respectively. The experimental results show that the sparse logistic regression method with *L*_1/2_ penalty is superior to the three existing regularization methods.

**Table 4:** Precision and recall results for sparse logistic regression with *L*_1/2_ and *L*_1_ penalties, Ridge Regression and Elastic Net in the combined band dataset.

**Method**	**Neutral**		**Positive**		**Negative**	
	**Precision**	**Recall**	**Precision**	**Recall**	**Precision**	**Recall**
*L*_1/2_	**0.86** ± **0.01**	**0.87** ± **0.01**	**0.90** ± **0.05**	**0.88** ± **0.02**	**0.86** ± **0.03**	**0.86** ± **0.03**
*L*_1_	0.82 ± 0.04	0.79 ± 0.06	0.88 ± 0.01	0.88 ± 0.01	0.86 ± 0.07	0.79 ± 0.01
Ridge Regression	0.83 ± 0.01	0.87 ± 0.01	0.88 ± 0.04	0.88 ± 0.01	0.75 ± 0.05	0.79 ± 0.01
Elastic Net	0.81 ± 0.02	0.83 ± 0.02	0.90 ± 0.02	0.81 ± 0.05	0.78 ± 0.01	0.83 ± 0.03

*Neutral, Positive and Negative are Emotional categories. Precision and Recall are are the criteria for judging the quality of the method. Numbers indicate the best of the four results*.

Supplementary Figure 4 shows an accuracy box plot for the different methods obtained from the 5-fold cross-validation. It can be seen from the box plot that better results were obtained with sparse logistic regression with the *L*_1/2_ penalty than with the *L*_1_ penalty or Ridge Regression, or with the Elastic Net method. The fluctuation range for sparse logistic regression with the *L*_1/2_ penalty was not large, demonstrating that the proposed method is more stable than the others. Thus, even in the worst case, it is superior to the other methods.

**Figure 4:**
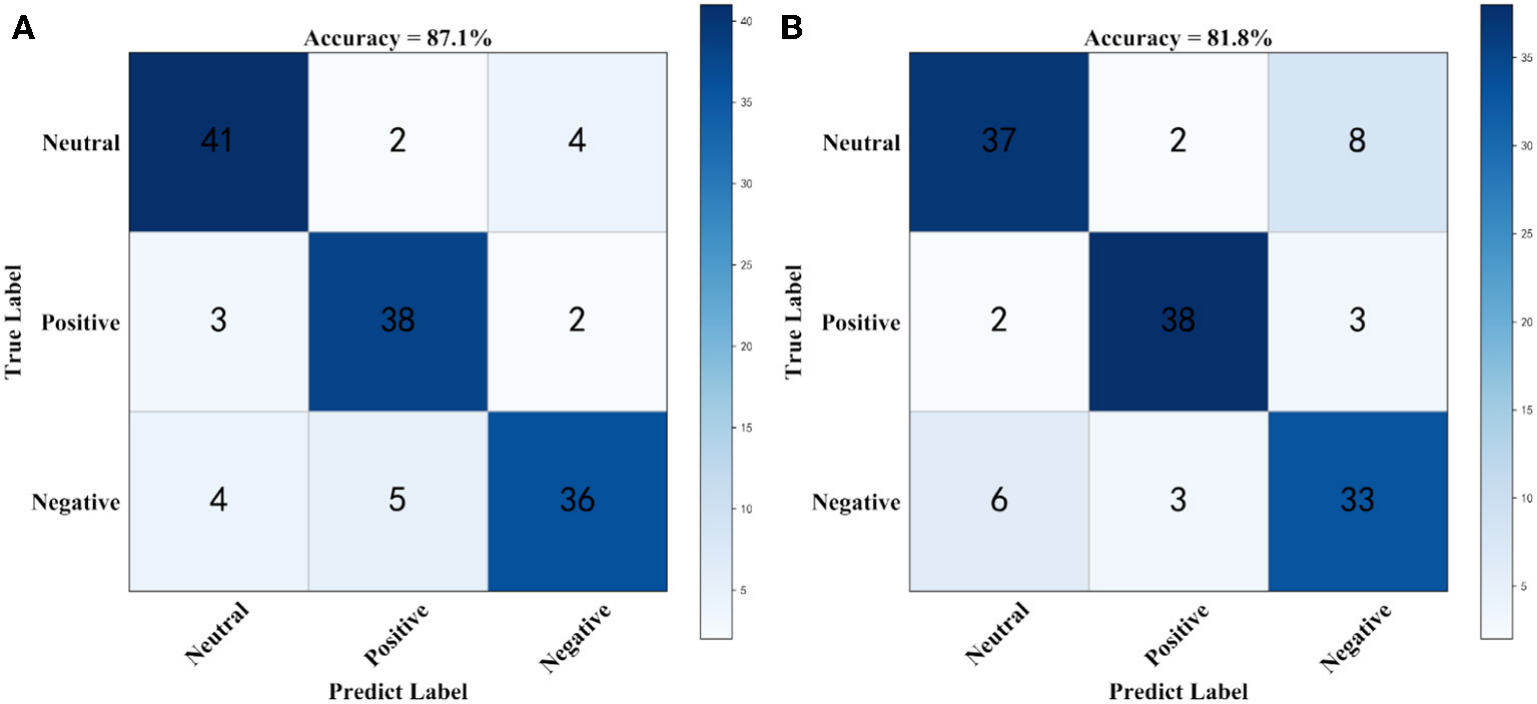
The Confusion matrix using combined band dataset. **(A)** Confusion matrix for generating prediction results using sparse logistic regression with *L*_1/2_ penalty, **(B)** Confusion matrix for generating prediction results using sparse logistic regression with *L*_1_ penalty.

Figure 4 shows the confusion matrix for generating prediction results using sparse logistic regression with the *L*_1/2_ and *L*_1_ penalties for the two datasets. As shown in Figure 4, the accuracy of the sparse logistic regression method with *L*_1/2_ penalty was 87.1%, and the results obtained with the proposed method were significantly better than those for sparse logistic regression with the *L*_1_ penalty or Ridge Regression, or the Elastic Net method (Supplementary Figure 5). In the SEED combined band dataset, there are 30 labels that were predicted incorrectly using the sparse logistic regression method with *L*_1/2_ penalty with the main errors concentrated in the Negatives class. However, compared with the sparse logistic regression method with *L*_1/2_ penalty, the number of incorrect labels is four fewer. Thus, it can be concluded that sparse logistic regression with the *L*_1/2_ penalty was the most accurate method for classification of the EEG emotion recognition dataset.

In the SEED-combine dataset, similar conclusions are obtained in this paper. As can be seen from the Table 5, the *L*_1/2_ penalty retained only 111, 111, and 140 data points in the three binary classifiers, while the *L*_1_ method retained 214, 163, and 22 data points, respectively. Ridge regression and Elastic Net retained all data points. The *L*_1/2_ penalty retained the fewest data points but gave the best results. It shows that the *L*_1/2_ penalty used in this paper has excellent sparsity in EEG sentiment data.

**Table 5:** The results of Number of points retained after sparsification by the sparse Logistic regressions with *L*_1/2_, *L*_1_ penalties, Ridge Regression, and Elastic Net in combined band dataset.

**Method**	**Classifier 1**	**Classifier 2**	**Classifier 3**
*L*_1/2_	**111**	**111**	**140**
*L*_1_	214	163	226
Ridge Regression	323	323	323
Elastic Net	323	323	323

*Neutral, Positive and Negative are Emotional categories. Precision and Recall are are the criteria for judging the quality of the method. Numbers indicate the best of the four results*.

### 3.4. Analysis of DEAP Dataset

As can be seen from Table 6, in the SEED dataset, the logistic regression based on the *L*_1/2_ penalty proposed in this paper obtained the best results. In terms of the average accuracy, the result of the logistic regression method based on *L*_1/2_ penalty reached 71%. Compared to the Ridge Regression and Elastic Net methods, it has improved by 1%. Compared to *L*_1_ penalty, it is increased by 3%. In terms of standard deviation, the logistic regression of *L*_1/2_ penalty has the smallest fluctuation, showing excellent stability.

**Table 6:** The results of accuracy, precision and recall by the sparse Logistic regressions with *L*_1/2_, *L*_1_ penalties, Ridge Regression, and Elastic Net in DEAP dataset.

**Method**	**Positive**		**Negative**		**Accuracy**
	**Precision**	**Recall**	**Precision**	**Recall**	
*L*_1/2_	**0.76** ± **0.02**	**0.79** ± **0.02**	**0.65** ± **0.03**	**0.67** ± **0.02**	**0.73** ± **0.01**
*L*_1_	0.73 ± 0.03	0.75 ± 0.03	0.60 ± 0.06	0.58 ± 0.02	0.68 ± 0.03
Ridge Regression	0.73 ± 0.02	0.71 ± 0.06	0.62 ± 0.03	0.62 ± 0.03	0.70 ± 0.013
Elastic Net	0.73 ± 0.02	0.71 ± 0.06	0.63 ± 0.03	0.65 ± 0.05	0.70 ± 0.012

*Neutral, Positive and Negative are Emotional categories. Precision and Recall are are the criteria for judging the quality of the method. Numbers indicate the best of the four results*.

Supplementary Figure 6 shows an accuracy box plot for the different methods obtained using 5-fold cross-validation. It can be seen from the box plot that the results for the sparse logistic regression method with *L*_1/2_ penalty were significantly better than those of the other three methods.

Table 7 shows the number of loci retained by different methods after calculation. Among them, the *L*_1/2_ penalty performed very well, retaining only 301 sites, compared to the 1,537 sites retained by the *L*_1_ penalty. The *L*_1/2_ penalty retained 80.4% of the sites, but the final result was better than the *L*_1_ penalty. Experimental results show that the *L*_1/2_ penalty has the best sparsity in the EEG dataset, *L*_1_ has poor sparsity, and Ridge Regression and Elastic Net do not have sparsity.

**Table 7:** The results of Number of points retained after sparsification by the sparse Logistic regressions with *L*_1/2_, *L*_1_ penalties, Ridge Regression, and Elastic Net in DEAP dataset.

**Method**	**Classifier 1**
*L*_1/2_	**301**
*L*_1_	1,537
Ridge Regression	2,082
Elastic Net	2,082

*Neutral, Positive and Negative are Emotional categories. Precision and Recall are are the criteria for judging the quality of the method. Numbers indicate the best of the four results*.

## 4. Discussion

In EEG sentiment classification, only a small portion of the EEG signal strongly indicates the individual's emotional state. Therefore, feature selection methods play an important role. In this paper, we propose a sparse logistic regression model based on the *L*_1/2_ penalty and develop the corresponding coordinate descent algorithm as a new EEG feature selection method. The proposed method uses a new univariate half threshold to update the estimated coefficients. In typical regularization methods, the sparsity of the *L*_0_ penalty is theoretically the best; however, this method is difficult to solve, making it less practical. The *L*_1_ penalty and Ridge Regression are theoretically mature and are commonly used. However, neither has good enough sparsity. There are regularizers that are sparser than the *L*_1_ penalty, such as *L*_1/2_ (Xu et al., 2010, 2012). Our results suggest that for EEG emotion datasets, higher sparsity leads to finding features from the EEG signal that are more relevant and have better predictive power for emotion classification.

The *L*_1/2_ penalty has been used with very good results in many fields, including image processing and genetic data (Liang et al., 2013; Liu et al., 2014; Huang et al., 2016). This method is very suitable for small-sample, high-dimensional data, such as emotion-based EEG datasets. The existing public datasets typically contain small samples with high dimensions and high levels of noise. If all EEG signals are included in the calculations, the algorithm will have high computational complexity and be prone to overfitting. Therefore, in this work, we used the *L*_1/2_ penalty combined with logistic regression and proposed a new three-class sparse logistic regression model. The results demonstrate that the logistic regression method based on the *L*_1/2_ penalty performs better than other regularization methods. That is, the sparseness of the *L*_1/2_ penalty in the EEG emotional dataset was better than those of the *L*_1_ penalty and Ridge Regression. Simulation and real data experiments showed that sparse logistic regression with the *L*_1/2_ penalty achieved higher classification accuracy than the conventional *L*_1_, Ridge Regression, and Elastic Net regularization methods. Therefore, sparse logistic regression with *L*_1/2_ penalty is an effective technique for EEG sentiment classification. To verify the sparsity of the *L*_1/2_ penalty logistic regression in EEG sentiment dataset, we count the first five EEG Positions retained by the *L*_1/2_ penalty in two datasets (Supplementary Tables 1, 2). Based on data from brainmaster magazine (company) and existing articles (Larsen et al., 2000; Jordan et al., 2001; Lin et al., 2010; Zheng and Lu, 2015). In SEED dataset, all electrode points, including FP1, F8, FPZ, FT8, and FP2 are related to Experiencing/processing emotion. In the DEAP dataset, among them FC5, FC6, CP6, and PO4 Positions are related to Experiencing/processing emotion. The AF3 Position is related to Fear response. Combining the *L*_1/2_ penalty in the experiment, yielded the least points of all methods. This shows that the *L*_1/2_ penalty has good sparsity for EEG sentiment data. It may also mean that the AF3 Position and EEG processing emotion are highly correlated. In this work, we only combined the *L*_1/2_ penalty with the logistic regression method and did not consider combinations including the latest brain networks or deep learning methods (Bernal et al., 2019). Therefore, further work is needed. However, we believe that our proposed approach complements existing sparse methods for EEG emotional data classification well (Wang et al., 2019), which will help researchers to better analyze such data.

## Data Availability Statement

Publicly available datasets were analyzed in this study. This data can be found here: http://bcmi.sjtu.edu.cn/~seed/index.html and http://www.eecs.qmul.ac.uk/mmv/datasets/deap/.

## Ethics Statement

Written informed consent was obtained from the individual(s) for the publication of any potentially identifiable images or data included in this article.

## Author Contributions

D-WC and RM proposed the method. Z-YD conducted the experiments. Y-YL, YL, and LH read and modified the manuscript. All authors contributed to the manuscript and approved the submitted version.

## Conflict of Interest

The authors declare that the research was conducted in the absence of any commercial or financial relationships that could be construed as a potential conflict of interest.
