# Supplementary material for: Sparse Logistic Regression With L1/2 Penalty for Emotion Recognition in Electroencephalography Classification
Source: Front Neuroinform. 2020 Aug 7;14:29. doi: 10.3389/fninf.2020.00029 (PMC7427509; doi:10.3389/fninf.2020.00029)
Supplement: Supplementary file 1 [file Data_Sheet_1.pdf]

Supplementary Material

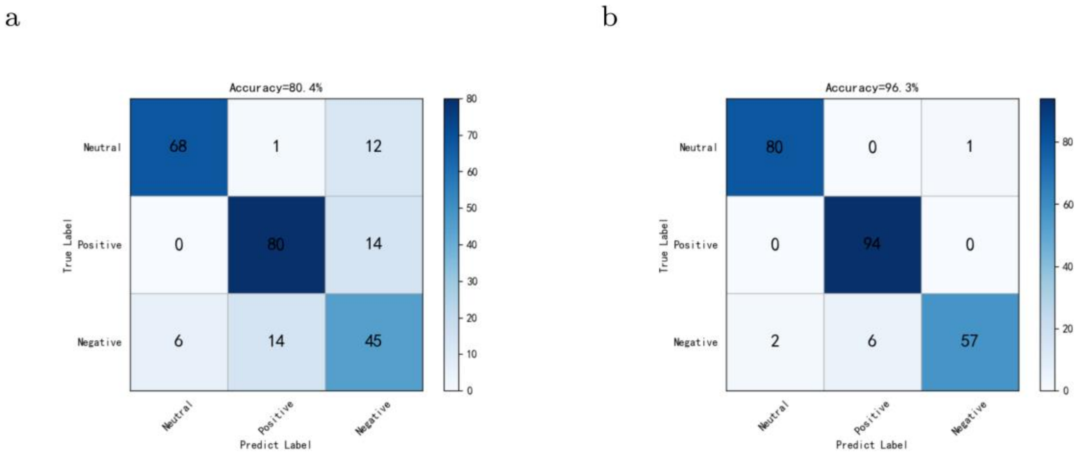

Supplementary Figure 1. The Confusion matrix using simulation dataset.
(a)Confusion matrix for generating prediction results using Ridge Regression,
(b)Confusion matrix for generating prediction results using Elastic Net.

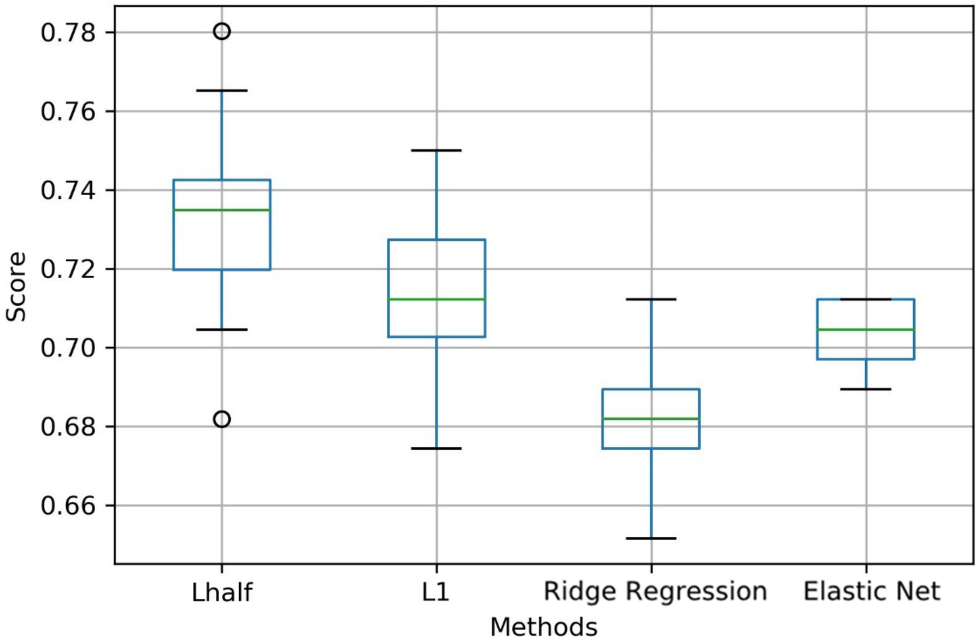

Supplementary Figure 2. The accuracy of box plot of different methods in the case of five-fold cross-validation in Beta band dataset. From the figure we can see that the $L_{1/2}$ penalty logistic regression method proposed in this paper has achieved the best and stable results. The second is the L_1 penalty logistic regression method. The worst is ridge regression.

a b

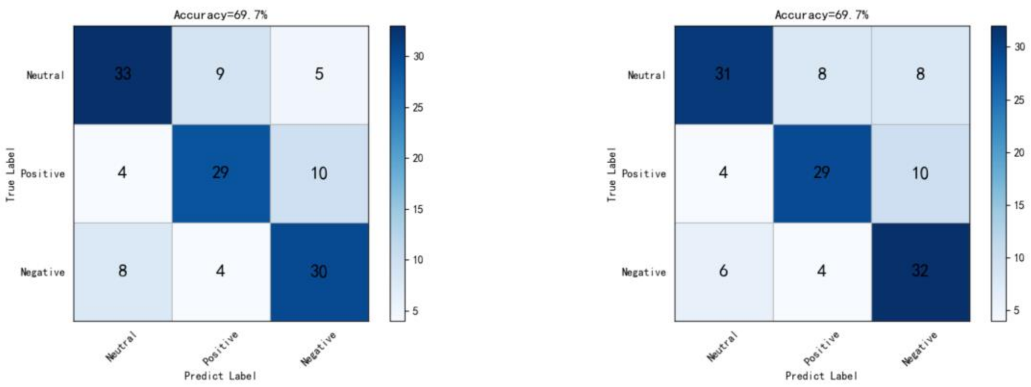

Supplementary Figure 3. The Confusion matrix using Beta band dataset.
(a)Confusion matrix for generating prediction results using Ridge Regression,
(b)Confusion matrix for generating prediction results using Elastic Net.

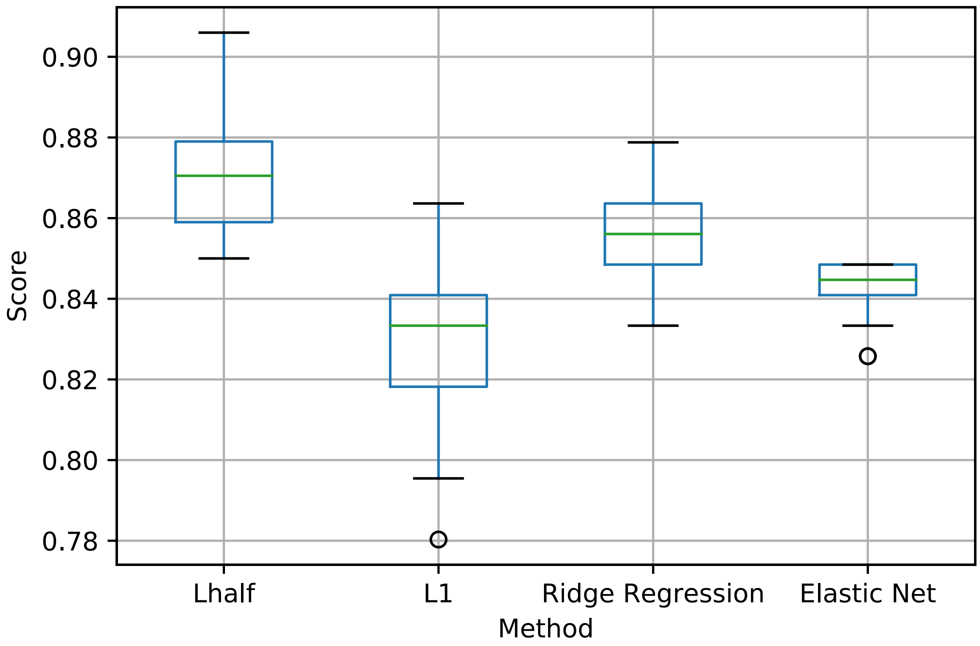

Supplementary Figure 4. The accuracy of box plot of different methods in the case of five-fold cross-validation in combined band dataset. From the figure we can see that the $L_{1/2}$ penalty logistic regression method proposed in this paper has achieved the best and stable results. The second is the ridge regression method. The worst is the L_1 penalty logistic regression method.

a b

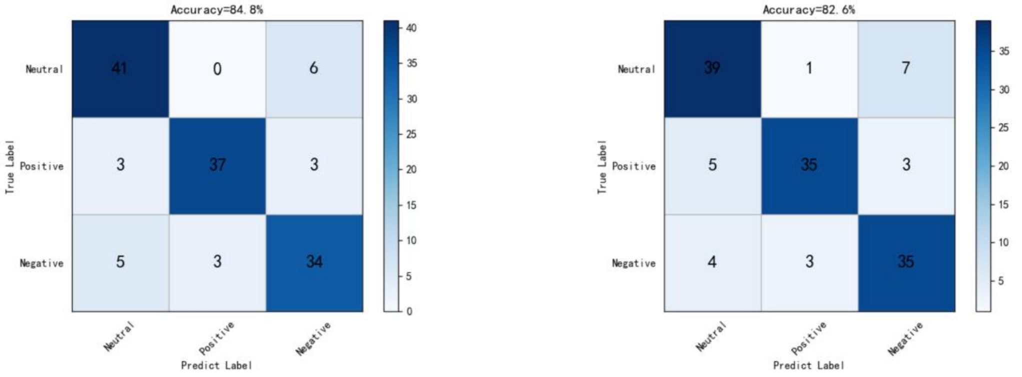

Supplementary Figure 5. The Confusion matrix using combined band dataset.
(a)Confusion matrix for generating prediction results using Ridge Regression,
(b)Confusion matrix for generating prediction results using Elastic Net.

Supplementary Table 1 Top 5 EEG Positions found in the SEED dataset using sparse logistic regression with $L_{1/2}$ penalty

Positions
FP1
F8
FPZ
FT8
FP2

Supplementary Table 2 Top 5 EEG Positions found in the DEAP dataset using sparse logistic regression with $L_{1/2}$ penalty

Positions
AF3
FC5
FC6
CP6
PO4

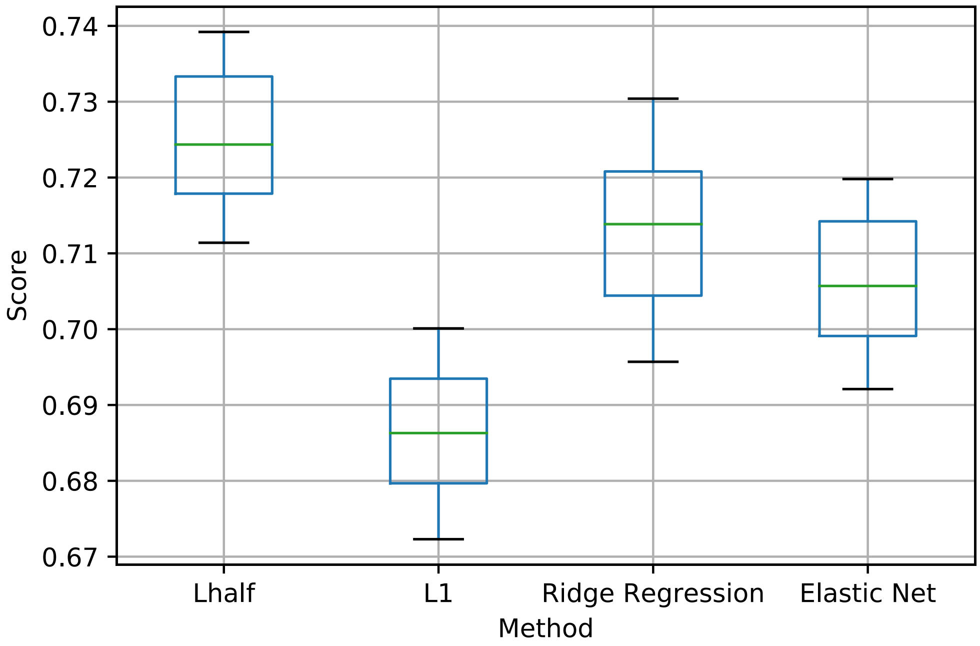

Supplementary Figure 6. The accuracy of box plot of different methods in the case of five-fold cross-validation in DEAP dataset. From the figure we can see that the $L_{1/2}$ penalty logistic regression method proposed in this paper has achieved the best and stable results. The second is the ridge regression method. The worst is the L_1 penalty logistic regression method.
